# A pilot feasibility study comparing large language models in extracting key information from ICU patient text records from an Irish population

**DOI:** 10.1186/s40635-024-00656-1

**Published:** 2024-08-16

**Authors:** Emma Urquhart, John Ryan, Sean Hartigan, Ciprian Nita, Ciara Hanley, Peter Moran, John Bates, Rachel Jooste, Conor Judge, John G. Laffey, Michael G. Madden, Bairbre A. McNicholas

**Affiliations:** 1 Department of Anaesthesia and Intensive Care Medicine, University Hospital Galway, Galway, H91 YR71 Ireland; 2 School of Computer Science, University of Galway, Galway, Ireland; 3 Anaesthesia and Intensive Care Medicine, School of Medicine, University of Galway, Galway, Ireland; 4 School of Medicine, University of Galway, Galway, Ireland

**Keywords:** Large language models, Medical summaries, Intensive care medicine, Artificial intelligence

## Abstract

**Background:**

Artificial intelligence, through improved data management and automated summarisation, has the potential to enhance intensive care unit (ICU) care. Large language models (LLMs) can interrogate and summarise large volumes of medical notes to create succinct discharge summaries. In this study, we aim to investigate the potential of LLMs to accurately and concisely synthesise ICU discharge summaries.

**Methods:**

Anonymised clinical notes from ICU admissions were used to train and validate a prompting structure in three separate LLMs (ChatGPT, GPT-4 API and Llama 2) to generate concise clinical summaries. Summaries were adjudicated by staff intensivists on ability to identify and appropriately order a pre-defined list of important clinical events as well as readability, organisation, succinctness, and overall rank.

**Results:**

In the development phase, text from five ICU episodes was used to develop a series of prompts to best capture clinical summaries. In the testing phase, a summary produced by each LLM from an additional six ICU episodes was utilised for evaluation. Overall ability to identify a pre-defined list of important clinical events in the summary was 41.5 ± 15.2% for GPT-4 API, 19.2 ± 20.9% for ChatGPT and 16.5 ± 14.1% for Llama2 (*p* = 0.002). GPT-4 API followed by ChatGPT had the highest score to appropriately order a pre-defined list of important clinical events in the summary as well as readability, organisation, succinctness, and overall rank, whilst Llama2 scored lowest for all. GPT-4 API produced minor hallucinations, which were not present in the other models.

**Conclusion:**

Differences exist in large language model performance in readability, organisation, succinctness, and sequencing of clinical events compared to others. All encountered issues with narrative coherence and omitted key clinical data and only moderately captured all clinically meaningful data in the correct order. However, these technologies suggest future potential for creating succinct discharge summaries.

**Supplementary Information:**

The online version contains supplementary material available at 10.1186/s40635-024-00656-1.

## Background

Intensive care medicine necessitates the delivery of systematic, high-quality medical care alongside life-saving treatments. Artificial intelligence (AI) offers the promise of system improvements and enhanced resource allocation to optimise ICU care delivery [1, 2]. AI-based algorithms, capable of predicting deteriorating patient outcomes and mortality using extensive datasets, have gained traction [3, 4].

Large language models (LLM) such as ChatGPT, GPT-4 API and Llama2 can interrogate and summarise large volumes of medical notes to create succinct summaries of radiology reports, internal medical progress notes and patient dialogue [5–8]. Their use in clinical practice remains in the development stage due to data protection and limitations of the technology. Although recognised as a paradigm-changing technology, significant limitations exist with LLMs including the generation of ‘hallucinations’ from the data [9]. Many of the studies on LLM have been conducted using large anonymised US datasets [5–7]. Studies using clinical data from European datasets are challenging due to ethical and legal concerns surrounding large-scale data processing with LLMs under current European laws. The General Data Protection Regulation (GDPR) mandates that individual patient consent is obtained for processing patient data, including when using commercially available LLMs. As a result, European studies often face limitations based on the number of patients from whom consent can be obtained. It is crucial to thoroughly assess the accuracy and safety of LLMs being introduced by electronic medical record (EMR) providers and software companies which are being deployed without the stringent testing that pharmaceuticals and medical devices typically require before widespread use [10]. This pilot feasibility study aims to address these challenges by investigated three commercially available LLM’s ability to accurately and concisely synthesise ICU discharge summaries focusing on their accuracy, readability, recall and completeness of critical information and presence of hallucinations, thereby determining the viability of LLMs in enhancing clinical documentation processes in ICU settings.

## Methods

### Ethics

Adult patients who were admitted to the ICU in Galway University Hospital who had capacity were approached for inclusion in the study and informed consent for participation was obtained. Informed consent was taken by an investigating doctor conducted in accordance with the ethical principles outlined in the Declaration of Helsinki. Ethical approval for the study was obtained from the Galway University Hospital Research Ethics Committee on 27/7/2023(C.A. 2973).

### Participants and dataset

We used clinical notes generated during each consecutive ICU admission. Notes were stored in an electronic health record system (Metavision^®^, Tel Aviv, Israel). Laboratory or radiology data were not included unless their findings were summarised in the clinical notes. The clinical notes were a combination of nurses’, doctors’, and pharmacists’ notes. The notes consisted of unstructured text, containing clinical terminology and abbreviations. The notes were divided into “sessions”, each of which was preceded by a header indicating the date and time of the entry. Before submission for processing by the LLM’s, the patient notes were fully anonymised by clinical staff and all personal identifiers were removed. Anonymisation of dates in each set of patient notes was performed by writing a program to subtract a random, fixed number of months from each date. This was necessary to ensure that the continuity of the timeline of the patient’s ICU stay was maintained.

### Large language models

The LLM’s tested were ChatGPT, GPT-4 API, and Llama 2. Rationale for choice of LLM is outlined in the supplementary appendix. ChatGPT currently uses OpenAI’s GPT-4 large language model [11]. The version released on August 3, 2023 was used throughout this study. For GPT-4 API, at the time of development, the latest model was gpt-4-0613 and this was used in all experiments. The context window length of the model was 8000 tokens. The Llama-2-70b-chat model with the HuggingFace inference API was used for testing the capabilities of this model at performing the summarisation task. Since patient notes may be longer than the input length limits of LLMs, for the analyses with GPT-4 and Llama 2, the Langchain framework [12] was used to split and notes into manageable lengths, process them, and recombine outputs. ChatGPT had no programming interface to enable it to be used with Langchain, so documents were submitted as a series of smaller chunks.

### Prompting and managing hallucinations

Alongside recent advancements in LLMs, prompt engineering has emerged as an effective, low-cost method of enhancing the quality of LLM outputs for specific tasks. Recent literature has investigated the application of prompt engineering to the healthcare domain, as a means of exploiting the potential of LLMs to extract information from large volumes of medical data [6, 21–23]. In this study, we used zero-shot prompting, whereby the prompt alone outlined the output requirements, without providing examples. To minimise creativity or diversity, temperature was set to zero or almost zero in the case of Llama 2 (see Supplementary Appendix). There was no limit of word count but instructions in prompts were to “generate concise summaries”. As noted in the Supplementary Appendix, we saw significant hallucinations in previous work [11], and in this work we carried forward prompting techniques to minimise them, such as prompting “Please ensure that the summary is based purely on information contained within the notes”, and by reducing temperature.

### Development and evaluation

The development and evaluation are outlined in supplementary Fig. 1. The notes of five episodes were used in the development phase and the notes of the remaining six episodes in the evaluation phase (Supplementary Fig. 1). The outputs from each iteration were analysed by the clinicians involved in the development process (BM, JR, SH), who provided feedback which guided improvements. Prompts were iteratively developed, beginning with a baseline version which outlined the basic requirements for the summaries. These included instructing the model to highlight key interventions and developments, to use language suitable for a medical doctor and to only include information contained within the notes. Five iterations were carried out in total. Each iteration entailed generating a summary of a specific episode, which was then reviewed by clinicians. The prompt was updated to address the feedback provided by the clinicians and the summary was re-generated to confirm that the requested improvements had been included. Any summaries generated in previous iterations were re-generated each time the prompt was modified to verify that the results had not been adversely affected. Clinicians identified extraneous information within summaries, which was resolved by requesting a “concise” summary within the prompt. To generate notes relevant to a healthcare provider subgroup (doctor/nurse/pharmacy), prompts were generated stating that their notes should be given precedence for inclusion and headers distinguished between the types of notes generated by each healthcare providers subgroup. After addressing the feedback for the five episodes used in development, the prompt was finalised and used to generate summaries of unseen patient notes during the evaluation phase.

In the evaluation phase, three consecutive runs for each set of patient notes on each LLM were analysed by the clinicians involved in the development process (BM, JR, SH). They selected the best one in each case for evaluation by independent blinded evaluators (RJ, PM, JB, JL, CH).

### Scoring

A checklist specifying essential information template for scoring LLM-generated summaries was developed from clinical notes by three investigators involved in the development process (BM, JR, SH). This was completed prior to evaluation of the generated LLM transcript. The scoring criteria included the presence of information and its correct placement within the summary.

Each summary were scored on their inclusion of a pre-defined number of relevant clinical events. Evaluators assigned scores based on the accuracy of reporting of these events: 1 point for properly noted events, 0.5 points for partially noted events, and 0 points for omitted events. Additionally, the placement of each clinical event was scored: 1 point for appropriate placement, 0.5 points for moderately appropriate placement, and 0 points for inappropriate placement. The scores for both inclusion and placement were totalled, divided by the maximum possible score, and then converted to a percentage. Readability, organisation, succinctness, and accuracy were assessed using a five-point Likert scale, with 1 indicating the lowest and 5 the highest quality. Evaluators ranked the LLM summaries with 1 being the best and 3 being the worst. Definitions for readability, organisation, succinctness and accuracy of reporting and instructions for are outlined in supplementary appendix. Evaluators, who were not involved in LLM transcript generation, were provided with the checklist, all the patients’ data used to generate LLM transcripts, and patients’ chart numbers, so clinical data generated by LLM could be verified. Finally, a free-text column in which overall opinion on the summary was collected. Each evaluator was responsible for evaluating the 3 selected outputs for each of two sets of patient notes. The outputs for each set of patient notes were evaluated by two independent evaluators.

### Statistical analysis

Quantitative variables are reported using either the mean and standard deviation (SD) for normally distributed data or the median and interquartile range (IQR) for non-normally distributed data. The non-parametric Kruskal–Wallis test was utilised for the comparison of ordinal and rank data. The Kappa statistic was calculated to assess interrater reliability between evaluators. Statistical significance was established at a p-value threshold of less than 0.05.

## Results

The study was conducted between July 2023 and September 2023 and utilised clinical details from 11 ICU episodes in 9 patients (5 female, 4 male). Demographics, reason for admission, length of stay are outlined in Table 1. Patient length of stay ranged from 3 to 73 days and number of events for the LLM summaries to capture ranged from 7 to 22 consistent with the level of complexity of the admission. Most admissions involved medically complex patients and three of the cohort subsequently died during their hospital admission. Word count of data submitted for summary generation ranged from 325 to 26699 for transcripts used to develop prompts and 15021 to 61006 for transcripts used for evaluation. The LLM summary word counts ranged from 98 to 740 (Table 1).

**Table 1:** Description of ICU patients used for development and evaluation of LLMs for summarising ICU clinical notes

Episode number	Patient number	Age	Gender	ICU LOS	Reason for admission	Number of clinical events for assessment in summaries	Transcript word count	LLM note word count
1	1	76	F	6 days	Electrolyte abnormalities	Development	3614	NA
2	2	70	F	3 days	Type 2 respiratory failure	Development	3257	NA
3	3	28	M	26 days	Road traffic accident with massive haemorrhage and intra-abdominal sepsis	Development	3273	NA
4	4	67	F	4 days	Post 2 stage oesophagectomy	Development	26699	NA
5	6–2	69	M	4 days	Neutropenic sepsis	Development	16343	NA
6	6–1			15 days	New diagnosis DLBCL with transverse myelitis	17	25662	GPT4- 561 ChatGPT-490 Llama2-171
7	9	70	M	17 days	COVID pneumonitis	16	21865	GPT4- 462 ChatGPT-452 Llama2-98
8	7	69	F	5 days	Post hemicolectomy	7	15021	GPT4- 236 ChatGPT-601 Llama2-414
9	5–1	51	F	14 days	Respiratory sepsis	18	15075	GPT4- 416 ChatGPT-509 Llama2-417
10	5–2			41 days	Intra-abdominal sepsis	22	38379	GPT4- 740 ChatGPT-359 Llama2-415
11	8	67	M	73 days	Cerebellar stroke with failure to wean from ventilator	20	61006	GPT4- 664 ChatGPT-310 Llama2-310

^*^DLBCL diffuse large B-cell lymphoma, *LOS* length of stay

**Fig. 1:**
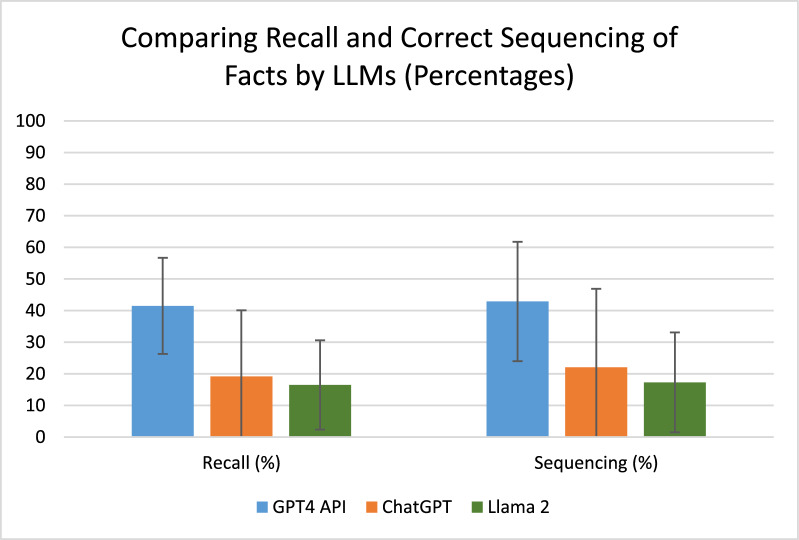
Comparison of recall and correct sequencing of clinical details by LLMs in summarising intensive care unit clinical notes

Overall ability to recall a pre-defined list of important clinical events in the summary was 41.5 ± 15.2% for GPT-4 API, 19.2 ± 20.9% for ChatGPT and 16.5 ± 14.1% for Llama (p = 0.002). Appropriate sequencing of facts, an indicator of the LLMs ability to appropriately rank clinically significant events was highest for GPT-4 API (42.9 ± 18.9%) compared to 22.1 ± 24.8% for ChatGPT and 17.3 ± 15.8% for Llama (*p* = 0.009) (Fig. 1, Table 2).

**Table 2:** Summary of LLMs ability to summarise ICU clinical notes

	ChatGPT^™^	GPT-4 API	Llama 2^™^	*P*-value
Readability	3 ± 1	3.3 ± 0.5	2.2 ± 0.8	0.07^b^
Organisation	2.5 ± 1.1	2.9 ± 1.0	1.7 ± 0.6	0.016^b^
Succinctness	2.5 ± 1	2.9 ± 1	1.5 ± 0.8	0.018^b^
Accuracy	1.8 ± 1.2	2.3 ± 1.3	1.3 ± 0.5	0.25^b^
Rank	2 ± 0.6	1.2 ± 0.4	2.8 ± 0.4	0.01^b^
Inclusion of parameters (%)	19.2 ± 20.9	41.5 ± 15.2	16.5 ± 14.1	0.002
Order of appearance in text (%)	22.1 ± 24.8	42.9 ± 18.9	17.3 ± 15.8	0.009

^a^Rank 1 best, 3 worst

^b^Kruskal–Wallis test

Readability, organisation, succinctness, accuracy: 1 is worst, 5 is best

**Fig. 2:**
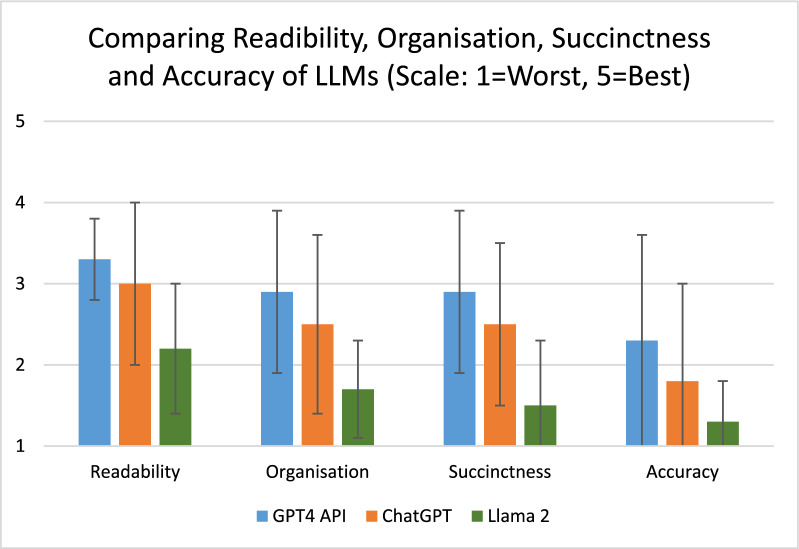
Comparison of readability, organisation, succinctness, and accuracy of LLMs in summarising intensive care unit clinical notes

Of the three different LLMs used to generate a summary for each patient episode, GPT-4 had significantly higher scores for organisation, and succinctness and was non-significantly higher for readability and accuracy compared to ChatGPT (Fig. 2, Table 2). GPT-4API was ranked the best (1.2 ± 0.4) followed by ChatGPT (2.0 ± 0.6) and Llama 2 had consistently lowest score for rank (2.8 ± 0.4), *p* = 0.1. Llama2 had the lowest score for all parameters (Table 2). Summary of feedback for each LLM is noted in Table 4, with Llama 2 being noted to have generic and repetitive summaries that did not capture all clinical events. GPT-4 API and ChatGPT were noted to have good readability but omitted clinical events.

Excerpts of anonymised LLM summaries and the list of clinical events from which they were benchmarked are reported in the supplementary appendix. Overall, comparing reviewers’ opinions (Table 3), there was moderate agreement for readability and rank, and low agreement for succinctness and accuracy (both related to what is included) and organisation of text (related to the order of appearance of events).

**Table 3:** Interrater reliability

	Kappa statistic	p-value
Readability	0.397	0.006
Organisation	0.126	0.3
Succinctness	− 0.154	0.3
Accuracy	0.254	0.09
Rank	0.417	0.01

Hallucinations were noted in GPT-4 API summaries only and there were four in total. These are outlined in Table 4 and of minor clinical significance. No hallucinations were identified in our analyses of the outputs of ChatGPT or Llama 2. The outputs of ChatGPT and Llama 2 were less comprehensive, as reflected in the quantitative data of Table 2 and the free-text comments of Table 4; since they included fewer potentially factual statements in their outputs, they had a lower propensity to hallucinate.

**Table 4:** Free text feedback from evaluators and examples of hallucinations on LLM

	GPT-4 API	ChatGPT^™^	Llama 2^™^
Negative	“Lack of coherent storytelling” “Omission of crucial details” “Inaccuracies such as implying the patient was on vasopressors at discharge”	“Left out several details” “Lacked pertinent clinical information” “Inaccurately described clinical events- errors regarding organ failure and life support”	“Absence of crucial clinical events failure to mention significant therapeutic interventions, while noting minor ones generic and repetitive statements
Positive	“Better alignment of key events and a well-organized structure”	Logical organisation. Easy readability	
Examples of hallucination	1.Vasopressin and noradrenaline to be discontinued after ICU discharge 2. Propofol and dexmedetomidine to be discontinued after ICU discharge 3.Blood transfusion for low platelets 4.Patient not for central line although patients had central line placed and was to be discharged with central line	No hallucinations	No hallucinations

## Discussion

In this study, we evaluated the efficacy of three LLMs in generating accurate, organised, and succinct ICU discharge summaries. Our analysis revealed that the GPT-4 API outperformed ChatGPT and Llama 2 in terms of readability, organisation, succinctness, and the accurate sequencing of clinical events. Although GPT-4 API was the preferred model, it still exhibited issues such as a lack of narrative coherence and omissions of key clinical data. Overall, none of the LLMs could identify more that 40% of events considered by trained intensivists to be important, with major differences between open source and commercially available LLM providers. This assessment underscores the varying capabilities of LLMs in handling complex medical data and highlights the challenges in achieving optimal accuracy and coherence in automated discharge summaries.

The optimal means to evaluate the quality of LLM summaries has yet to be established. Our benchmark for assessing LLMs was based on a list of key clinical events highlighted by physicians rather than comparing LLM summaries with physician generated summary of clinical events. As our study was on critical care patients, subject to numerous interventions and managed by various healthcare professionals, we focused on the clinical events in which the patients underwent rather than creating an ideal expert summary. Although a list is similar to a summary, summaries emphasise readability and style over content which is captured in a list. In health systems that are not billing based, the documentation focus of hospital notes is on event recording for peer communication and medico-legal reasons, with less emphasis on billing purposes (public payer system). Most LLM studies have depended on human lead semi-quantitative assessment of coherence, comprehensiveness, harmfulness and factual inconsistencies as well as comparing LLM with human based summaries [5, 13, 14]. Automated metrics do not correlate with quality and human input to score coherence, inconsistencies, comprehensiveness and harmfulness are semi-quantitative and will differ based on the use care in which LLM are applied [5, 13].

In addition to testing LLMs’ ability to refer to the listed clinical events, we also tested the systems’ ability to emphasise their clinical implications based on where they were placed in the text, i.e. in a testing scenario administration of routine electrolytes was mentioned before a patient being on a naloxone infusion for opiate overdose. Overall, we found that while summaries had reasonable score for readability, their ability to list all clinically relevant events was only moderate and this is consistent with other studies that found that error rates increased with greater length of texts [13]. LLMs’ ability to generate summaries that have logical clinical information is consistent with that of recent studies where adjudicators observed that AI-generated notes often lacked clinical logic, a predictable outcome considering that AI is based on statistical likelihood of subsequent words rather than deductive reasoning [15]. In contrast, in a study from University of Florida [14], two physicians assessed clinical paragraphs produced by a GPT architecture and those written by UF Health physicians. The evaluation criteria included readability, clinical relevance/consistency, and the ability to discern if the text was AI or physician generated. Results showed similar linguistic readability and clinical relevance across both sets of notes, with physicians unable to reliably identify whether notes were AI or physician generated.

Overall, hallucinations were of minor clinical significance because the prompts directed the use of only existing data. This restriction might have limited the LLMs ability to accurately incorporate clinical events into the summary. Outputs were shorter and less complex than the checklist generated for intensivists to score against, showing that recognition and prioritising medically important issues needs optimisation. Balancing creativity and only using data present may lead to summaries that are not able to link all data points necessary to be inputted into a summary. Understanding the implications of bias introduced by prompt structures leading LLMs to generate outputs where none exist needs to be understood [16]. Further honing of prompts may improve this in future iterations, but overall, further work is needed in assessing the safety and comprehensiveness of LLM-generated summaries before they are incorporated into clinical practice.

There are several limitations of this study, the first being limited size. Due to General data protection regulation (GDPR) legislation, we required individual patient consent for data processing. Although a consent waiver could have been requested, there was a need to establish a scientific merit for this type of study to allow its approval. GDPR prohibits mass processing of individual patient data without consent and from this there was a legal and ethical requirement that limited us to include patients from whom we could obtain consent. We ensured anonymity by manually removing identifying information, as automated tools removed clinically relevant data. The study's limited size reflects these ethical and legal challenges, which also complicate large-scale data processing using commercial LLMs. Conducting larger studies in Europe poses significant challenges due to stringent legislation and Europe is notably underrepresented in scientific outputs related to clinical summaries using LLMs. Most studies applying LLM on clinical notes have been conducted in the US without individual consent, using de-identified data, with only one study from France utilising retrospective MRI reports without identifiable data [5, 6, 13, 14, 17–20]. This pilot study highlights the need for further research to explore the potential role of LLMs in clinical settings. It also suggests that legislative changes and increased funding are necessary to allow safe and ethically appropriate access to patient records, particularly free text notes, for research purposes. Such advancements are crucial for leveraging technology to improve patient care and advance medical research.

Other limitations of this study include the longer-term clinical relevance of these findings given the speed of development in this field, its inclusion of only health care provider generated text without laboratory and radiological results, and the application of GPT4-API generated prompts to the other LLM rather than independently generated prompts separately for each model. It is possible that the performance of the LLMs in recalling key events (but not the sequencing of these events) was restricted by the need to produce concise summaries of a very large amount of clinical information. We did not compare the summaries generated by LLM with those created by physicians. Instead, we used a comprehensive checklist detailing relevant clinical events. It is possible that these events might not be included in a physician’s summary, depending on their writing standards. This approach provided a more robust gold standard for evaluating the LLM, especially in medical practices where billing does not rely on physician documentation. It is worth noting that we designed the evaluation criteria after the prompt development phase. It may be possible to further improve the prompts so that outputs would be more clearly aligned with the evaluation criteria. We propose to examine this in future work, and to evaluate newer LLM releases with longer context windows, as they become available. Finally, we did not examine for bias related to ethnicity, known to affect outputs from LLM’s [21, 22], as the population in the North/Northwestern region of Ireland is over 90% Caucasian. However, we did include a balance of male and female participants.

In summary, LLM models can produce readable summaries from free text data generated during ICU admissions with GPT-4 API producing the best results compared to ChatGPT and Llama2. However these require further optimisation to ensure all clinically meaningful events are correctly documented before their widespread adoption in clinical medicine.

## Supplementary Information

Supplementary Material 1.

## Data Availability

Prompts and coding to generate data are available in the Appendix. Anonymised examples of output in Appendix.

## Abbreviations

AI: Artificial intelligence
API: Application programming interface
GPT: Generative pre-trained transformer
GDPR: General data protection regulation
ICU: Intensive care unit
LLaMA2: Large language model meta AI 2
LLM: Large language model

## References

[1] Lu Y, Wu H, Qi S, Cheng K (2023) Artificial intelligence in intensive care medicine: toward a ChatGPT/GPT-4 way? Ann Biomed Eng 51(9):1898–1903 37179277 10.1007/s10439-023-03234-w PMC10182840

[2] Komorowski M, Del Pilar Arias Lopez M, Chang AC (2023) How could ChatGPT impact my practice as an intensivist? An overview of potential applications, risks and limitations. Intensive Care Med 49(7):844–847 37256340 10.1007/s00134-023-07096-7

[3] Johnson AE, Pollard TJ, Shen L et al (2016) MIMIC-III, a freely accessible critical care database. Sci Data 3:160035 27219127 10.1038/sdata.2016.35 PMC4878278

[4] Institute PeR. eICU Collaborative Research Database. 2023. https://eicu-crd.mit.edu/about/eicu/. Accessed 19/09/2023.

[5] Van Veen D, Van Uden C, Blankemeier L et al (2024) Adapted large language models can outperform medical experts in clinical text summarization. Nat Med 30(4):1134–1142 38413730 10.1038/s41591-024-02855-5 PMC11479659

[6] Guevara M, Chen S, Thomas S et al (2024) Large language models to identify social determinants of health in electronic health records. NPJ Digit Med 7(1):6 38200151 10.1038/s41746-023-00970-0 PMC10781957

[7] Schwartz IS, Link KE, Daneshjou R, Cortes-Penfield N (2023) Black box warning: large language models and the future of infectious diseases consultation. Clin Infect Dis. 10.1093/cid/ciad633 37971399 10.1093/cid/ciad633 PMC11006107

[8] Patel SB, Lam K (2023) ChatGPT: the future of discharge summaries? Lancet Digit Health 5(3):e107–e108 36754724 10.1016/S2589-7500(23)00021-3

[9] O PA (2021) Shaking the foundations: delusions in sequence models for interaction and control. arXiv preprint. 10.4855/arXiv.2110.10819

[10] Center MN. https://news.microsoft.com/2023/04/17/microsoft-and-epic-expand-strategic-collaboration-with-integration-of-azure-openai-service/. 2023. Accessed May 1st 2023.

[11] Madden MG, McNicholas BA, Laffey JG (2023) Assessing the usefulness of a large language model to query and summarize unstructured medical notes in intensive care. Intensive Care Med 49(8):1018–1020 37338549 10.1007/s00134-023-07128-2

[12] Python.langchain.com. Langchain: MapReduce. 2023. https://python.langchain.com/docs/modules/chains/document/map_reduce. Accessed 19/09/2023.

[13] Tang L, Sun Z, Idnay B et al (2023) Evaluating large language models on medical evidence summarization. NPJ Digit Med 6(1):158 37620423 10.1038/s41746-023-00896-7 PMC10449915

[14] Peng C, Yang X, Chen A et al (2023) A study of generative large language model for medical research and healthcare. NPJ Digit Med 6(1):210 37973919 10.1038/s41746-023-00958-w PMC10654385

[15] Boussen S, Denis JB, Simeone P, Lagier D, Bruder N, Velly L (2023) ChatGPT and the stochastic parrot: artificial intelligence in medical research. Br J Anaesth 131(4):e120–e121 37516646 10.1016/j.bja.2023.06.065

[16] Monica Agrawal SH, Hunter Lang, Yoon Kim, David Sontag. Large Language Models are Few-Shot Clinical Information Extractors. 2022. Accessed 1st May 2024.

[17] Williams CYK, Bains J, Tang T et al (2024) Evaluating large language models for drafting emergency department discharge summaries. medRxiv. 10.1101/2024.04.03.24305088 39484284 10.1101/2024.10.15.24315548 PMC11527060

[18] Williams CYK, Zack T, Miao BY et al (2024) Use of a large language model to assess clinical acuity of adults in the emergency department. JAMA Netw Open 7(5):e248895 38713466 10.1001/jamanetworkopen.2024.8895 PMC11077390

[19] Chuang YN, Tang R, Jiang X, Hu X (2024) SPeC: a soft prompt-based calibration on performance variability of large language model in clinical notes summarization. J Biomed Inform 151:104606 38325698 10.1016/j.jbi.2024.104606 PMC11608453

[20] Le Guellec B, Lefevre A, Geay C et al (2024) Performance of an open-source large language model in extracting information from free-text radiology reports. Radiol Artif Intell. 10.1148/ryai.230364 38717292 10.1148/ryai.230364 PMC11294959

[21] Zack T, Lehman E, Suzgun M et al (2024) Assessing the potential of GPT-4 to perpetuate racial and gender biases in health care: a model evaluation study. Lancet Digit Health 6(1):e12–e22 38123252 10.1016/S2589-7500(23)00225-X

[22] Wang J, Shi E, Yu S, Wu Z, Ma C, Dai H, Yang Q, Kang Y, Wu J, Hu H, Yue C (2023) Prompt engineering for healthcare: methodologies and applications. arXiv preprint. 10.4855/arXiv.2304.14670

[23] Meskó B (2023) Prompt engineering as an important emerging skill for medical professionals: tutorial. J Med Internet Res 25:e50638 37792434 10.2196/50638 PMC10585440

