# Supplementary material for: A pilot feasibility study comparing large language models in extracting key information from ICU patient text records from an Irish population
Source: Intensive Care Med Exp. 2024 Aug 16;12:71. doi: 10.1186/s40635-024-00656-1 (PMC11327225; doi:10.1186/s40635-024-00656-1)
Supplement: Supplementary file 1 — Supplementary Material 1. [file 40635_2024_656_MOESM1_ESM.docx]

**Selection of LLM**

**GPT-4 API:** GPT-4 API was chosen because it was considered the most powerful publicly available LLM. It was built on the same technology as ChatGPT and had an application programming interface (API) that provided greater control over the model and its responses than ChatGPT. The GPT-4 API enabled the development of custom applications which interface with OpenAI’s GPT-4 LLM. Code developers had more control over the model and its responses than ChatGPT. For example, it is possible to control the determinism or “creativity” of responses by setting the value of the temperature parameter in the API. To ensure minimal creativity, or diversity, the temperature was set to zero. Hallucinations were only found in GPT-4 API and were not of clinical significance.

**ChatGPT:** ChatGPT was chosen as the most widely used LLM for advanced applications, and the one most likely to be used by clinicians in their work. It was more accessible to non-technical audiences than direct interaction with the API because of its user-friendly web interface. As a result of these considerations, it served as the initial testing environment during the development phase.

**Llama 2:** Llama 2 was chosen because, at that time, it was the most powerful open-source LLM available within Europe. Llama 2 is an open-source large language model, introduced by Meta and Microsoft in July 2023 (1). A collection of pretrained and fine-tuned variations of Llama 2 were available, ranging in size from 7 billion to 70 billion parameters (2). For the use case examined in this paper, the Llama-2-70b-chat model was determined to be the most suitable. The context window length of this model is 4000 tokens. Its ability to handle user and system prompts, in addition to its scale of 70 billion parameters made it the best candidate for comparison with the other models. The open-source nature of this language model could enable it to be run locally, given the availability of sufficient computational resources. Use of proprietary models such as ChatGPT and GPT-4 raises confidentiality concerns because of the requirement to upload data to third-party servers, where privacy cannot be guaranteed. Such concerns are blocking factors when dealing with highly sensitive patient data. Use of a locally run model (such as Llama 2) would eliminate the requirement to export patient data and therefore would mitigate these concerns. Identical prompts were used with minor tweaking to account for lack of API in ChatGPT. It is possible that different models respond differently to different prompts, it would have introduced additional complexity to the analysis if different prompts were used for each systems as each variation would have required testing in each system.

**Supplementary Appendix**

Development of prompts involved prompting ChatGPT™ to summarize a set of patient notes according to certain instructions. The length of the patient notes often exceeded the accepted input length of ChatGPT™, the integration of the GPT4 API with Langchain was devised as a solution to this problem. This has been addressed in subsequent iterations of LLM. This approach additionally provided control over the temperature parameter. A framework called Langchain was used to handle arbitrarily long inputs as at the time LLMs had a maximum context window that was shorter than the amount of text needed to be processed for a given patient case. The MapReduce chain operates by carrying out a “map” phase, followed by a “reduce” phase (Fig X). In the map phase, several documents are processed individually by a language model. The outputs from the map phase are then combined to form the input to the reduce phase. In the reduce phase, the language model is prompted to accurately combine the outputs from the map phase into a single output. This was the final output from the overall MapReduce chain. To further ensure uniformity across outputs, a template specifying the output format is defined within the prompt. Furthermore, in the MapReduce chain, the chunk size was set to 6000 tokens, the chunk overlap was set to 0 and the separator was defined as the newline character. As dates of key developments and interventions in the notes were required to be listed, the code programmatically appended the last session header in a chunk to the top of the next chunk. This meant that if the notes were split before the end of a chunk was reached, the corresponding time and date information was not lost.

In initial work((3)) we saw significant hallucinations, but minimised them by including prompts such as “Please ensure that the summary is based purely on information contained within the notes”, and by reducing temperature. In the case of Llama2™ 2, the required adjustments included setting temperature to a positive float as close to zero as the model would accept (1x), because a value of zero is not supported. The maximum output length was also required to be defined and was set to 1000 tokens. A smaller chunk size of 2000 tokens was required due to the shorter context length of Llama2™ 2 (4000 tokens) compared to GPT4 (8000 tokens).

In the case of ChatGPT™, integration with Langchain was not possible and the prompts were required to be submitted sequentially. Long documents were submitted as series of smaller chunks along with appropriate accompanying prompts. The initial prompt defined the output format and requirements, tagging the requested output type as an “ICUSum.” The patient notes were subsequently submitted with prompts asking for intermediate summaries. Once all chunks had been submitted, an “ICUSum” was requested to be generated.

**Development of Prompts for API-based models (GPT-4 API and Llama2)**

Prompts were defined using system and user prompts. System prompts are fixed instructions, provided to define the behaviour of the model during each phase. A single system prompt is supplied for the map phase, with a different one supplied for the reduce phase. The user prompt is prepended to each of the text chunks as they are submitted to the model. The final user and system prompts for the map and reduce phases are defined as follows:

1.  System prompt (Map phase)

“You are a medical analysis assistant, whose purpose is to generate a concise summary of the medical notes of a patient who was in ICU. The medical notes are a combination of doctors' and nursing notes. The nursing notes can be identified by containing the text: "Primary Nurse". Doctors' notes are of greater significance and should have greater influence on the contents of the summary. The summary will be read by a medical doctor. Please ensure that the summary is based purely on information contained within the notes. If significant information on the patient's background or admission to ICU is present in the notes, it should be included. Key interventions and key developments should be recorded, noting the date, time and reason for each. If the patient's post-ICU care plans are mentioned, record them. If mentioning antibiotics, state their names. If anticoagulants have been administered, state the name and dosage as this is important clinical information.”

2.  User prompt (Map phase)

“Here are the medical notes. Please review them carefully and generate a summary:

"(4)"”

    3.  System prompt (Reduce phase)

“You are a medical analysis assistant, whose purpose is to generate a concise summary of the medical notes of a patient who was in ICU. The summary will be read by a medical doctor. Please ensure that your summary is based purely on information contained within the notes. If mentioning antibiotics, state their names. If anticoagulants have been administered, state the name and dosage as this is important clinical information. Ensure the timeline of key developments and interventions is in chronological order.

Strictly adhere to this format:

## Patient Background and Admission to ICU ##

<Brief description of patient's age, gender, medical history and details of admission to ICU (only if specified).>

## Key Developments and Interventions ##

<- dd/mm/yyyy: concise description of key development/intervention and the reason for the development/intervention>

## Plans for Follow Up Care Post-ICU ##

<Concise summary of post-ICU care plans (if specified). Include essential information only>”

4.  User prompt (Reduce phase)

“Here are a set of summaries of medical notes recorded during the patient's ICU stay. Please review them carefully and generate an overall summary reflecting the key information from the intermediate summaries.

**Development of Prompts for ChatGPT**

ChatGPT required a different set of prompts than the API-based models (GPT-4 and Llama2), because it is not available via an API and can only be accessed via the web interface. Therefore, we were unable to use the Langchain MapReduce system with ChatGPT™. We aimed to replicate the workflow as accurately as possible using the following prompts:

1.  Initial prompt:

“You are a medical analysis assistant, whose purpose is to generate a concise summary of the medical notes of a patient who was in ICU, called an "ICUSum". The medical notes are a combination of doctors' and nursing notes. The nursing notes can be identified by containing the text: "Primary Nurse". Doctors' notes are of greater significance and should have greater influence on the contents of the summary. The summary will be read by a medical doctor. Please ensure that the summary is based purely on information contained within the notes. If significant information on the patient's background or admission to ICU is present in the notes, it should be included. Key interventions and key developments should be recorded, noting the date, time and reason for each. If the patient's post-ICU care plans are mentioned, record them. If mentioning antibiotics, state their names. If anticoagulants have been administered, state the name and dosage as this is important clinical information. Ensure the timeline of key developments and interventions is in chronological order. Strictly adhere to this format:

## Patient Background and Admission to ICU ##

<Brief description of patient's age, gender, medical history and details of admission to ICU (only if specified).>

## Key Developments and Interventions ##

<- dd/mm/yyyy: concise description of key development/intervention and the reason for the development/intervention>

## Plans for Follow Up Care Post-ICU ##

<Brief summary of post-ICU care plans (if specified). Include essential information only>

Are you ready?”

2.  Prompt supplied with the first chunk of notes:

“Thank you. Here is part 1 of the patient notes. Please review the notes carefully and provide an ICUSum: pasted chunk of notes here}”

3.      Prompt supplied with all chunks of notes, except the first and last chunks:

“Thank you. Continuing notes for the same patient. Here is part 4. Please review the notes carefully and provide an ICUSum:

{pasted chunk of notes here} ”

4.      Prompt supplied with the last chunk of notes:

“Thank you. Continuing notes for the same patient. Here is part 5. This is the last set of notes for the same patient. Please review parts 1 to 5 of the notes which I have provided carefully and provide an ICUSum:

{pasted chunk of notes here} ”

Evaluation

Due to the non-deterministic nature of LLMs, each generated output is different. Although this effect was minimized as much as possible in the case of the GPT4 API and Llama2™ 2 by minimizing the value of temperature, it was still necessary to account for this variability.

**Excerpts of transmission**

**Full transcripts of LLM summaries are not reported as consent for publication was not obtained. Excerpts from each LLM summary are presented which have been altered from actual summaries to ensure anonymization but are consistent with the reporting structure generated by the LLM.**

**GPT-4 API:**

Patient Background and Admission to ICU

The patient is a XXXXXX with a history of ****cancer treated with surgery, chemotherapy, and radiotherapy XX years prior. He was admitted to the ICU on XXXX due to acute shortness of breath and poor exercise tolerance. CT scans revealed a new lung malignancy causing bronchial obstruction and compression of bronchi and pulmonary arteries. The patient also had a pericardial effusion, for which a pericardial drain was placed. The patient also has a history of AFIB and was under the care of a nephrologist.

Key Developments and Interventions

• Patient admitted to ICU due to acute respiratory distress and poor exercise tolerance. Pericardial drain placed for pericardial effusion.

• Patient started on high-dose dexamethasone IV and Augmentin for prophylaxis. Patient also on Dexdor for sedation and treated with AIRVO/CPAP.

• Patient diagnosed with metastatic deposits of known ****cancer causing bronchial obstruction. Patient underwent first session of radiotherapy, planned for 10 sessions in total. Co-amoxiclav administered and **** stopped as per oncology's advice.

• Patient still intubated and ventilated. Patient on amiodarone infusion for paroxysmal atrial fibrillation and tazosin for possible lower respiratory tract infection. Plan for extubation after completion of radiotherapy.

• Patient still intubated and on Propofol. Patient still on amiodarone for paroxysmal atrial fibrillation. Patient had a positive fluid balance of +3.2L. Patient also on dexamethasone, which may be a risk factor for gastric ulceration.

**ChatGPT**

ICU Summary

Patient Overview: The patient has been in the ICU and recently transferred to HDU (High Dependency Unit), receiving consistent monitoring and interventions for multiple complications. They have been undergoing numerous sessions from 01/12/2019 to at least 04/12/2019.

Neurological Status: The patient's GCS fluctuated between 14 and 15 throughout the observation period, displaying confusion with regard to time and place initially but later showing improved orientation.

Respiratory Status: The patient has been self-ventilating, utilizing nasal specs and maintaining oxygen saturation on room air during the latest observations.

Cardiovascular Status: The patient experienced episodes of atrial fibrillation (Afib), with heart rates swinging between 85 to 142 bpm. Treatments included administration of medications like amiodarone and bisoprolol. They have been monitored for sinus rhythms and maintained a stable blood pressure.

**Llama2**

Based on the information provided in the medical notes, the patient has a history of AFIB and cancer. The patient is also at risk of re-feeding.

Based on the information provided in the medical notes, the patient was taking bisoprolol, thiamine, and lactulose. The patient was also given olanzapine or haloperidol as first-line agents.

Based on the information provided in the medical notes, the patient's plan for follow-up care post-ICU included a review of their medications, clarifying the anastrazole decision, and telemetry on the ward as the patient had a history of AFIB. The patient was also scheduled to be discharged to the ward.

Based on the information provided in the medical notes, the patient's vital signs included a blood pressure of 118/70, a heart rate of 104, a respiratory rate of 20, and a temperature of 36.4.

**Excerpt from Clinical decision list**

|  | Included | In suitable order | comments |
| --- | --- | --- | --- |
| 1.       Cause for admission and relevant background- malignancy with bronchial obstruction, severe shortness of breath and previous **** cancer treated with surgery/chemo and radiotherapy |  |  |  |
| 2.       Pericardial effusion |  |  |  |
| 3.       Placement of pericardial drain with loss of output, flumazenil administration |  |  |  |
| 4.       Worsening clinical status and proceeded to intubation for respiratory distress |  |  |  |
| 5.       Intubated and femoral line placed |  |  |  |

**Definition of categories for evaluators**

**Readability:** Score high for clear test without confusion, grammatically correct sentence structure, score low if ambiguous, confused, incorrect grammatical sentence structure

**Organisation:** Score high where clinically relevant events are highlighted over less relevant clinical events. Score low where less important clinical events highlighted over more relevant clinical events.

**Succinctness:** Score high where language is concise, clear, understandable sentences without unnecessary adjectives, score low where verbose, ambiguous and confusing.

**Accuracy:** Score high where overall it reflects the original content without distortion, error or hallucination. Score low where original content is distorted, inaccurate and incorrect.

**Rank:** A global score encompassing overall impression of each compared to each other with 1 being best, 3 being worst

**Inclusion of parameters:** Using the list of predefined clinical events for the summary assign 1 point if an event was properly noted, 0.5 points if it was partially noted, and 0 points if it was not noted.

**Order of appearance:** Using the list of predefined clinical events assign a score of 1 was given for appropriate placement, 0.5 for moderately appropriate placement, and 0 for inappropriate placement.

**Suplementary figures**


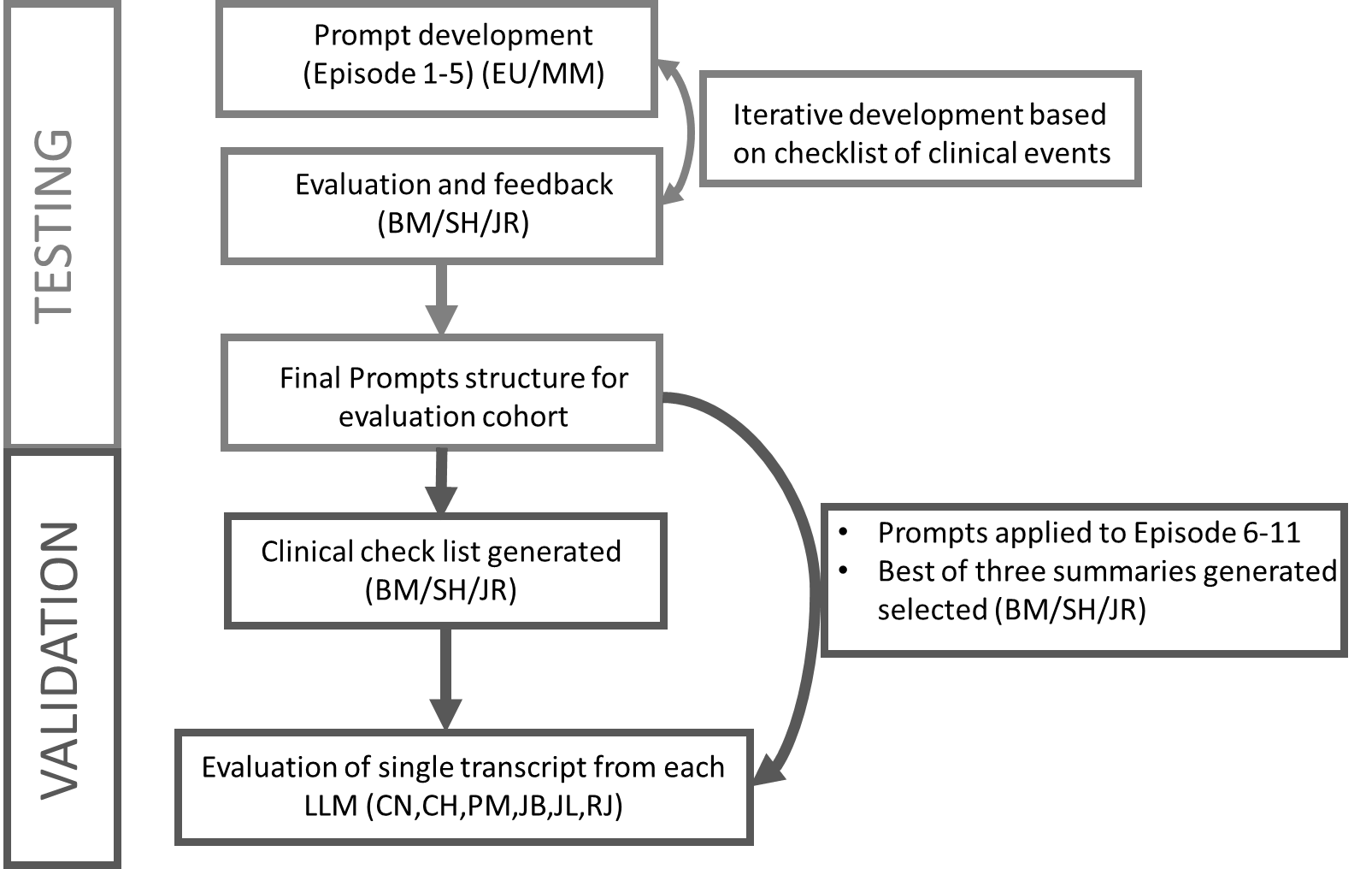


Supplementary Figure 1, Flow diagram: Outline of testing and evaluation phase.


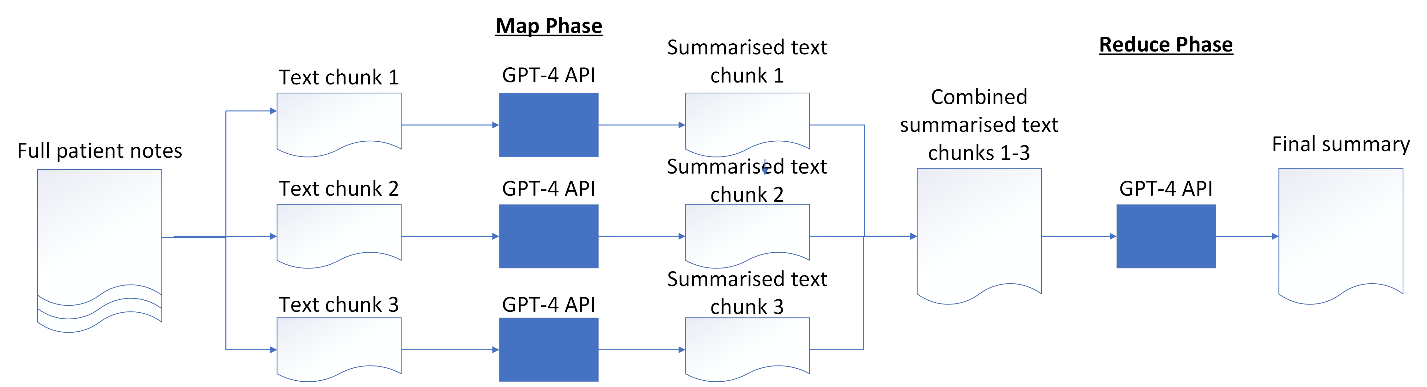


Supplementary Figure 2, *MapReduce workflow for patient record summarisation (using GPT-4 API as an example)*

References

1. Meta. Meta and Microsoft Introduce the Next Generation of Llama 2023 [Available from: <https://about.fb.com/news/2023/07/llama-2/>.

2. Touvron H. Llama 2: Open Foundation and Fine-Tuned Chat Models 2023 [Available from: <https://ai.meta.com/research/publications/llama-2-open-foundation-and-fine-tuned-chat-models/>

3. Madden MG, McNicholas BA, Laffey JG. Assessing the usefulness of a large language model to query and summarize unstructured medical notes in intensive care. Intensive Care Med. 2023;49(8):1018-20.

4. Dagci M, Cam F, Dost A. Reliability and Quality of the Nursing Care Planning Texts Generated by ChatGPT. Nurse Educ. 2023.
